# Waist-to-height ratio is better than body mass index and waist circumference as a screening criterion for metabolic syndrome in Han Chinese adults

**DOI:** 10.1097/MD.0000000000008192

**Published:** 2017-09-29

**Authors:** Hui Yang, Zhong Xin, Jian-Ping Feng, Jin-Kui Yang

**Affiliations:** a Department of Endocrinology, Beijing Tongren Hospital, Capital Medical University; b Department of Geriatric, Fu Xing hospital, Capital Medical University; c Beijing Key Laboratory of Diabetes Research and Care, Beijing, China.

**Keywords:** body mass index, metabolic syndrome, waist circumference, waist-to-height ratio

## Abstract

Metabolic syndrome (MetS) is a major public health concern. Efficient screening requires criteria that are economical, easily accessible, and applicable for all populations. We aimed to compare the discriminating ability of body mass index (BMI), waist circumference (WC), and waist-to-height ratio (WHtR) for the diagnosis of MetS in a Han Chinese population.

Demographic information, obesity indices, and results of biochemical tests were collected from a cross-sectional sample of 8084 individuals (3619 men and 4465 women, 18–79 years old) from Changping District, Beijing, China. Areas under receiver operating characteristic curves (AUCs) and adjusted odd ratios of 3 obesity indices were analyzed and their optimal cutoffs were determined.

For women, the AUCs demonstrated that WHtR was significantly more powerful than BMI and WC (both *P* *<* .05) for predicting MetS [WHtR, 0.857 (0.846–0.868); WC, 0.849 (0.837–0.860); BMI, 0.808 (0.795–0.821)]. For men, WHtR was significantly better than BMI [*P* *<* .05; WHtR, 0.859 (0.846–0.871); WC, 0.855 (0.843–0.868); BMI, 0.815 (0.802–0.829)]. The optimal cutoffs for WHtR for discriminating MetS were 0.51 in both genders. Multiple logistic regression confirmed the positive association between WHtR and the risk of MetS. In the nonobese subgroup, WHtR was also superior to BMI and WC for predicting MetS in men (*P* *<* .05) and better than BMI in women (*P* *<* .05).

Among the obesity indices analyzed here, WHtR was the best for predicting MetS in Han Chinese adults, especially in nonobese adults.

## Abstract 2

Supplemental Digital Content is available in the text

## 1 Introduction

Metabolic syndrome (MetS) is a constellation of metabolic risk factors that include abdominal obesity, hypertension, hyperglycemia, and dyslipidemia, the latter reflected by elevated triglycerides (TGs) and reduced high-density lipoprotein cholesterol (HDL-C).^[1]^ Individuals with MetS have a 5-fold higher risk of type 2 diabetes, 2-fold higher risk of cardiovascular disease (CVD), and overall higher risk of mortality than those without MetS.^[2,3]^ Alarmingly, the prevalence of MetS is increasing worldwide and is a major public health concern.^[4]^ China is the world's largest developing country and is experiencing an epidemic of MetS that is expected to continue into the near future.^[5]^ Therefore, a method to quickly diagnosis and control MetS will help relieve this social burden and reduce the prevalence of diabetes and CVD.

Several diagnostic criteria for MetS have been recommended. Body mass index (BMI) is not considered a valid criterion, because it does not reflect body fat distribution. Compared with BMI, measuring waist circumference (WC) is simple and inexpensive, yet effective for assessing body fat distribution and associated cardiovascular risk and mortality.^[6,7]^ In 2001, the National Cholesterol Education Program Adult Treatment Panel III proposed that high WC should be considered a feature of MetS. The American Heart Association/National Heart, Lung, and Blood Institute (AHA/NHLBI) and International Diabetes Federation (IDF)^[1]^ agreed that WC could be a useful primary screening tool for MetS diagnosis. However, recent studies have questioned the usefulness of WC, as it correlates closely with body-frame size. Therefore, the efficacy of WC for determining abdominal obesity is diminished for tall or short individuals.^[8,9]^ This is relevant for shorter populations including Asians, who were determined to have a greater prevalence of cardiometabolic risk factors than Caucasians, at the same WC value.^[10–12]^

Waist-to-height ratio (WHtR), as an indicator of central obesity, has been proposed as a better predictor of cardiovascular risk.^[13]^ Population-based studies have shown that WHtR is closely linked to cardiovascular risk, and receiver operating characteristic (ROC) curve analysis indicated a cutoff of 0.5 for Asian populations,^[14,15]^ as well as nonobese and normal-weight adults.^[16,17]^ However, relevant studies with Han Chinese urban residents have been scarce.

The present study investigated the comparative discriminative power of BMI, WC, and WHtR for predicting MetS in a group of Han Chinese adults, as well as a nonobese subgroup. The optimal cutoffs of WHtR were calculated and potential associations with age and gender were analyzed.

## 2 Methods

### 2.1 Study sample

The Medical Ethics Committee of Beijing Tongren Hospital approved the study protocol. All participants provided written informed consent to participate in this study. A cross-sectional, population-based survey on chronic diseases and risk factors was conducted from July 2010 to March 2011 in Changping district, Beijing. The survey covered an area of 1343.5 square kilometers and a permanent resident population of 1,660,500. Household sampling was performed by the Center for Disease Control and Prevention of Beijing. All residents were counted in each sampled household and 1 person was randomly recruited using Kish selection tables. Thus, 8155 eligible Han Chinese (ages, 18–79 years) were randomly selected from the households, and 21 declined participation. Potential enrollees with any of the following were further excluded: diagnosed cancer (n = 13); thyroid disease (n = 32); pregnancy (n = 2); skeletal deformities; amputation; or dependence on wheel chairs or other ambulatory assistive devices (n = 3). Finally, 8084 individuals (3619 men and 4465 women) were included in the present study.

### 2.2 Demographic data collection

The demographic data of each participant were collected via standardized questionnaire, covering disease history, smoking status, alcohol consumption, physical exercise, menopausal status, educational level, and family disease history. Disease histories included chronic conditions such as hypertension, diabetes, dyslipidemia. Smoking, drinking, physical exercise, and menopausal status were categorical variables defined as either “yes” or “no.” Smoking was defined as a total lifetime smoking of ≥100 cigarettes/cigars. Drinking was considered weekly consumption of ≥30 g of alcohol for ≥1 year. A “yes” response for physical exercise required ≥30 minutes of median-to-high intensity exercise ≥3 days per week. Educational levels included secondary, senior, college, and above; high educational level was defined as college and above.

### 2.3 Anthropomorphic measurements for obesity indices

Weight and height were taken by a standard measuring instrument, with each person wearing light clothing and without shoes. Weight was accurate to 100 g and height to 0.1 cm. BMI was then calculated as weight (kg) divided by height squared (m^2^). WC was measured at the midpoint between the lower edge of the rib cage and the iliac crest. WHtR was calculated as WC divided by height.

### 2.4 Clinical examination and biochemical tests

Blood pressure was measured thrice with the participant seated, at 5-minute intervals at the right arm using a standard mercury sphygmomanometer. The average of the last 2 measurements was adopted.

All participants underwent a comprehensive biochemical test, including lipid profiles and fasting plasma glucose (FPG) measurements. Twelve-hour fasting blood samples were collected for the determination of plasma glucose and lipids by auto-analyzer (Unicel DxC800; Beckman Coulter): TG, total cholesterol (TC), HDL-C, and low-density lipoprotein cholesterol (LDL-C).

Among all the participants, 3760 individuals were found with FPG ≥5.6 mmol/L. Of these, 2551 individuals successfully completed oral glucose tolerance tests. All participants who underwent an OGTT test were required to consume ≥150 g of carbohydrate food daily for 3 days before the test. On the day of the test, a total of 75 g of glucose powder in water was consumed by each participant and the blood sample was drawn 120 minutes after the consumption between 08:00 and 10:00 am. During the 120 minutes, each participant was asked to maintain normal physical activity. All specimens were analyzed within 24 hours.

### 2.5 Definitions

MetS was defined in accordance with the IDF and AHA/NHLBI,^[1]^ which requires ≥3 risk factors from the following 5 components: WC ≥80 cm in women or ≥90 cm in men; hypertension, considered as systolic blood pressure (SBP) ≥130 mm Hg or diastolic blood pressure (DBP) ≥85 mm Hg, or a prior diagnosis of hypertension with specific medication; hyperglycemia, defined as FPG ≥100 mg/dL (5.6 mmol/L) or a history of diabetes or anti-diabetic medication; TG ≥150 mg/dL (1.7 mmol/L) or relevant medication; and HDL-C <40 mg/dL (1.0 mmol/L) in men or <50 mg/dL (1.3 mmol/L) in women, or taking relevant medication.

In this study, a subgroup of individuals was considered nonobese on the basis of both BMI and WC. Normal BMI was defined as 18.5 to 23.9 kg/m^2^ and normal WC level was <90 cm for men and <85 cm for women.^[18,19]^

### 2.6 Statistical analysis

Statistical analyses were conducted using SPSS version 16.0 for Windows (SPSS, Chicago, IL) and MedCalc version 16.8 (http://www.medcalc.be). A *P* value < .05 was considered statistically significant. Continuous variables are shown as the mean ± standard deviation (SD), and categorical variables as case number and percentage. Comparisons between groups were performed using Student *t* test for continuous variables, and the Chi-squared test for categorical data. Partial correlation analysis was used to assess associations between the obesity indices and metabolic risk factors.

Adjusted odds ratios (ORs) and 95% confidence intervals (95% CIs) of 1-SD incremental increase in the obesity indices in association with MetS and its other components were calculated by multiple logistic regression analyses. Age, education level, smoking status, alcohol consumption, physical exercise, menopausal status (only women), and family history of the corresponding condition were used as confounders for multivariate analysis. ROC curves were plotted and analyzed using MedCalc version 16.8 (http://www.medcalc.be). Sensitivities and specificities were calculated and AUCs were compared to show the efficacy of the various obesity indices to discriminate subjects with and without MetS.

## 3 Results

### 3.1 Basic characteristics of the study sample

The genders were statistically similar in age and MetS prevalence (Table 1). Each of the obesity indices (BMI, WC, and WHtR) and the prevalence of hypertension were significantly higher in the men than in the women (all *P* < .001). Men also had significantly higher SBP and DBP; higher levels of FPG, TG, TC, and LDL-C; and lower levels of HDL-C, compared with the women (all *P* < .001).

**Table 1:**
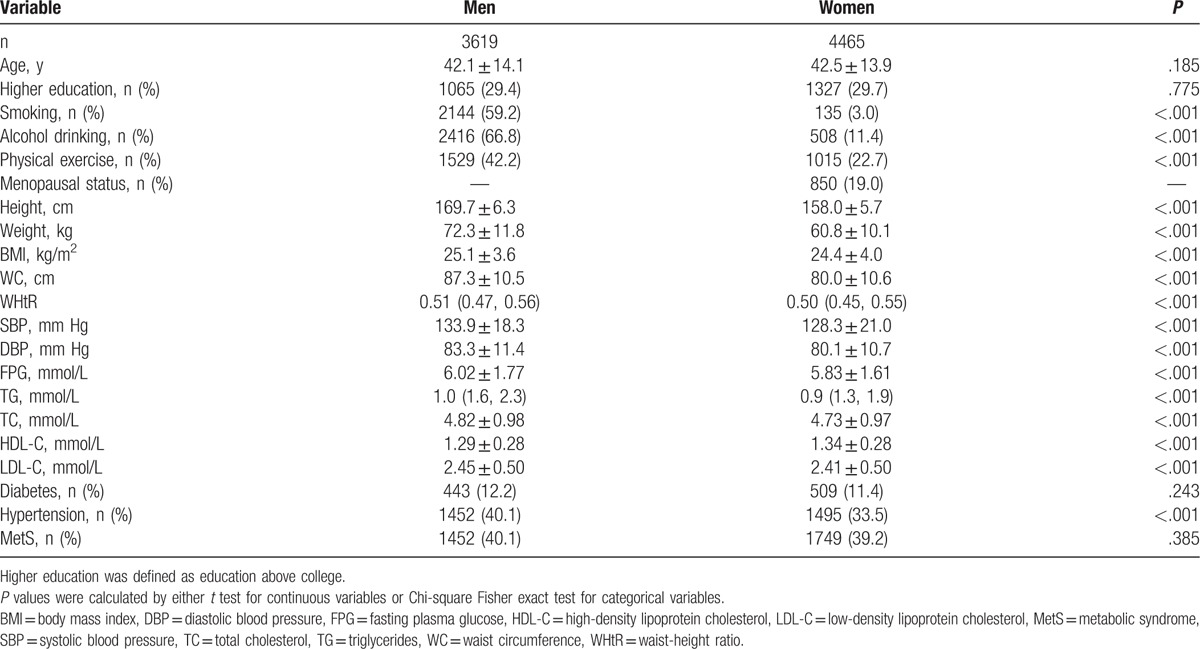
Basic characteristics of the study sample.

After adjusting for age, education level, smoking, alcohol drinking, and physical exercise, for both genders, the obesity indices (BMI, WC, and WHtR) were highly intercorrelated with each other, and they were also significantly correlated with metabolic risk factors, including hypertension, hyperglycemia, and dyslipidemia (all *P* < .05; supplementary Table S1).

### 3.2 Efficacy of WHtR, WC, and BMI in predicting MetS

**Figure 1:**
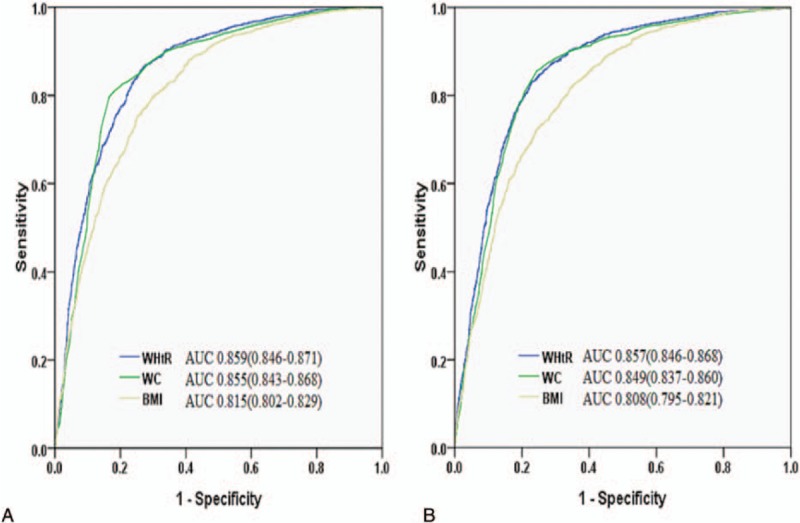
ROCs for body mass index (BMI), waist circumference (WC), and waist-to-height ratio (WHtR) for predicting MetS in men (A) and women (B). MetS = metabolic syndrome, ROC = receiver operating characteristic curve.

The discriminative efficacies of the obesity indices for predicting MetS in men or women were determined using ROC curves (Fig. 1; Table 2). For both genders, the AUC of the WHtR for predicting MetS overall was significantly higher than the AUCs of either the BMI or WC (both *P* *<* .05). Considering hypertension, hyperglycemia, and high TG, in both genders, the AUCs of the WHtR were significantly greater than that of BMI, except for lower HDL-C in men. Of note, in both genders, the AUCs of the WHtR were significantly greater than that of WC in predicting hypertension and hyperglycemia, and in predicting high TG in women (all *P* *<* .05; Table 2). Across all age groups, WHtR was also significantly superior to both BMI and WC in predicting MetS.

**Table 2:**
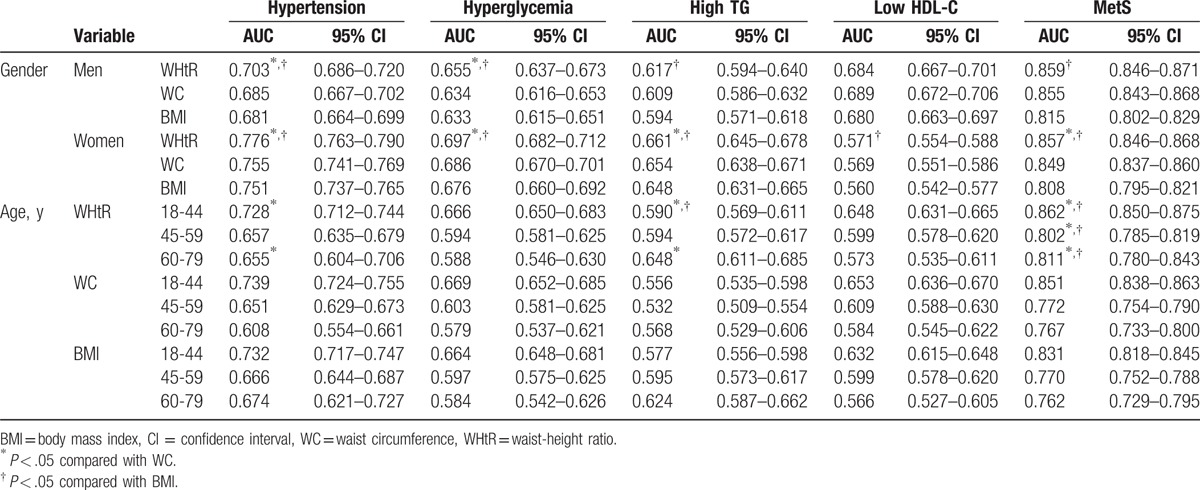
AUC (95% CI) for obesity indices for MetS and its components by gender and age ranges.

For both genders, the optimal cutoff value for the WHtR for MetS was 0.51 (Table 3). The cutoff for WC was 89.5 cm in men and 79.8 cm in women. The cutoff of BMI was 25.3 kg/m^2^ in men and 24.7 kg/m^2^ in women.

**Table 3:**
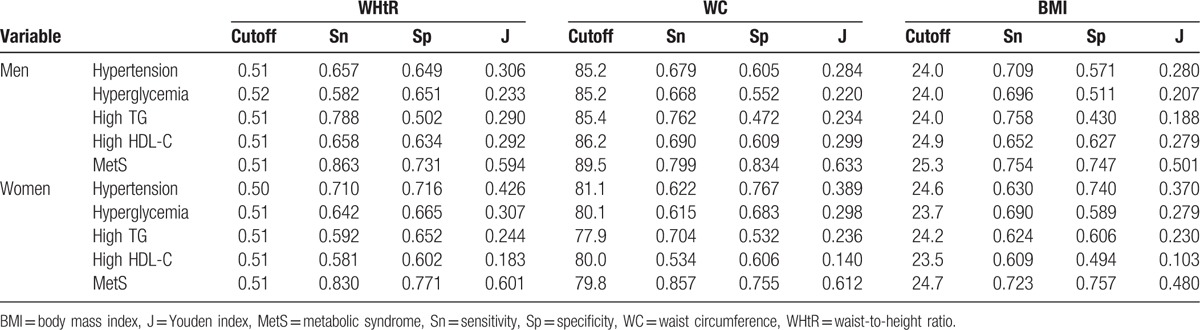
Optimal cutoffs of obesity indices determined by their sensitivities, specificities, and Youden indices for MetS and its components in total sample.

### 3.3 Associations between WHtR, WC, and BMI and MetS

In both men and women, after adjusting for confounding factors, all the obesity indices were significantly associated with the risk of MetS and its components (hypertension, hyperglycemia, high TG, and low HDL-C; all *P* < .05; Table 4). In men, each incremental increase in SD in WHtR increased the risk of MetS by 5.57 times (OR 5.57, CI 4.93–6.29, *P* *<* .05); an incremental increase in SD also significantly increased the risk of MetS for WC (OR 5.48, CI 4.86–6.18) and BMI (OR 4.31, CI 3.86–4.81; both *P* *<* .05). Similar results were also seen in women for WHtR (OR: 3.94, CI 3.52–4.40); WC (OR 3.76, CI 3.39–4.18; and BMI (OR 2.85, CI 2.59–3.13; all *P* *<* .05).

**Table 4:**
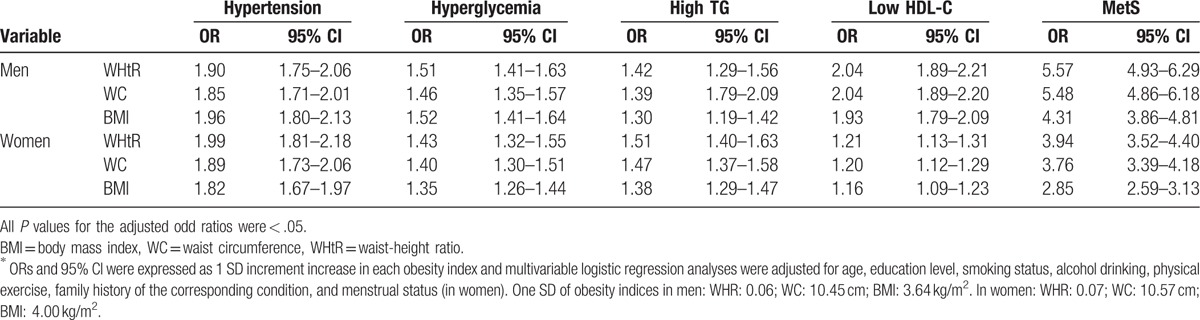
Adjusted ORs (95% CI) for MetS and its components in total sample^∗^.

### 3.4 Predicting MetS by obesity indices in the nonobese population

To elucidate further the usefulness of obesity indices in predicting MetS, we evaluated the ROC curves of the WHtR, WC, and BMI in predicting MetS in the nonobese subgroup (Table 5). A total of 3199 participants (1294 men and 1905 women) were defined as nonobese based on their BMI and WC. In men, the AUC of the WHtR was significantly higher than that of the WC and BMI in predicting MetS (all *P* *<* .05). In women, the AUC of the WHtR was significantly higher than that of the BMI (*P* *<* .05). The WHtR was also significantly superior to either WC or BMI in predicting hypertension and hyperglycemia in both men and women. No significant results were found regarding high TG in men or low HDL-C in women.

**Table 5:**
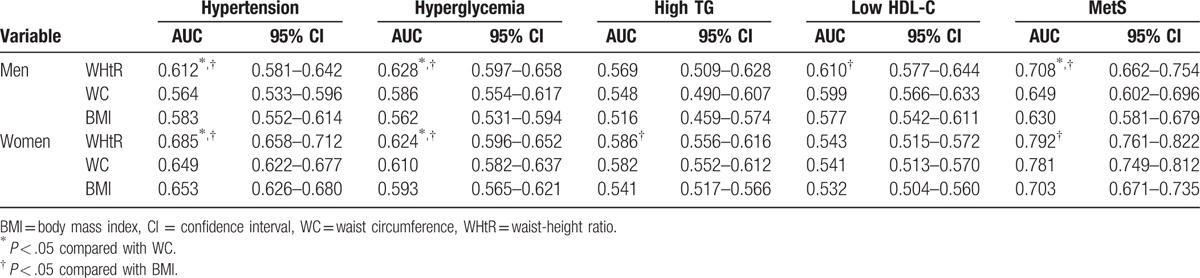
AUC (95% CI) for obesity indices for MetS and its other components in nonobese adults.

The optimal cutoff of the WHtR for predicting MetS was 0.48 for both men and women, whereas those for BMI and WC were 22.0 kg/m^2^ and 79.2 cm in men, and 22.2 kg/m^2^ and 77.4 cm in women, respectively (Table 6).

**Table 6:**
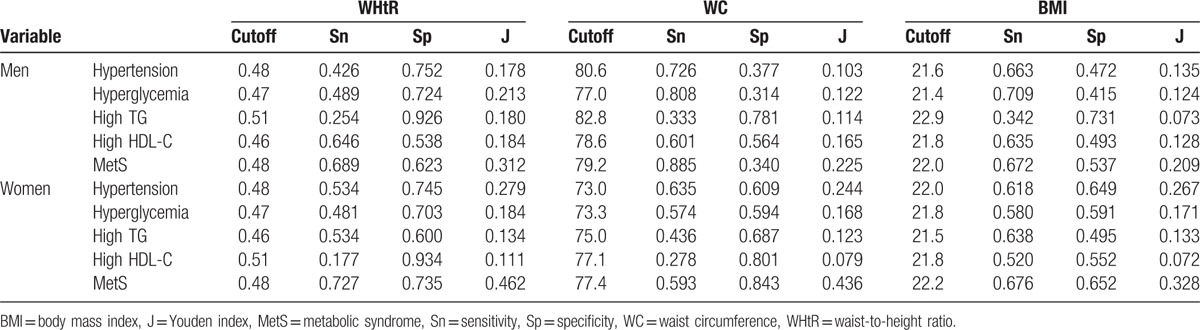
Optimal cutoffs of anthropometric indices and their sensitivities, specificities, and Youden indices for MetS and its components in nonobese adults.

After adjusting for confounding factors, all the obesity indices were significantly associated with an overall risk of MetS and each of its components (hypertension, hyperglycemia, high TG, and low HDL-C; all *P* < .05; Table 7). In men, similar findings were also found for WHtR, OR 1.77, CI 1.47 to 2.15; for WC, OR 1.63, CI 1.32 to 2.02; and for BMI, OR 1.61, CI 1.30 to 2.01; all *P* *<* .05. In women, each increase in SD in the WHtR increased the risk of MetS by 2.78 times (OR 2.78, CI 2.27–3.41; *P* *<* .05); in WC by 2.85 times (WC: OR: 2.85 (3.36–3.44); and BMI by 1.81 times (OR 1.81, CI 1.52–2.15; both *P* *<* .05).

**Table 7:**
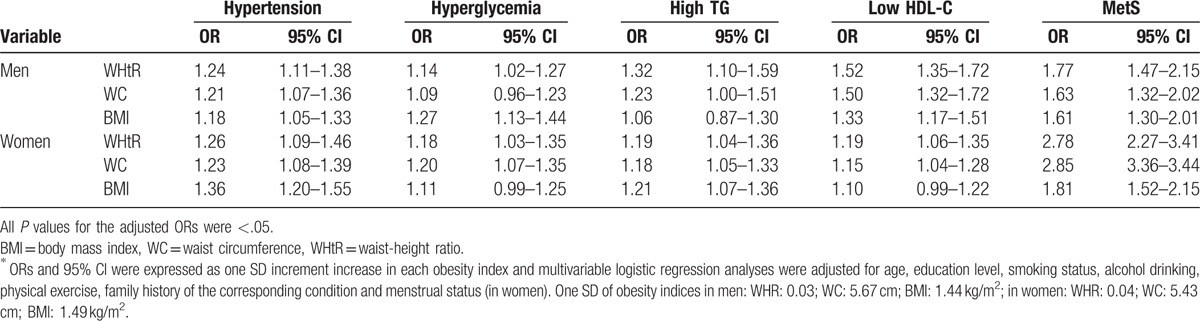
Adjusted OR (95% CI) for MetS and its components in non-obese adults^∗^.

## 4 Discussion

The present study showed that in a Han Chinese population, the WHtR can be more useful than either WC or BMI as a marker of MetS and other metabolic disorders. Furthermore, the subgroup defined as not obese based on BMI and WC was also susceptible to MetS, as indicated by higher WHtR. The data indicate that WHtR can be useful for identifying those in the Han Chinese population with MetS, especially for those who are not obese by the conventional definitions.

This study showed that, among the obesity indices measured, the WHtR was best at predicting MetS in the Chinese population. This is in accord with previous studies of Asians. For example, Chen et al^[20]^ also found that WHtR was better than BMI or waist-to-hip ratio in predicting MetS (based on the 2005 IDF criteria), although their cutoff value was slightly higher (0.55 in men and 0.56 in women) than ours. A similar trend was also found in another Chinese study, in which the superiority of WHtR over waist-to-hip ratio for predicting MetS was confirmed.^[21]^ Another study in Sri Lankan adults also showed that WHtR is superior to BMI, WC, or waist-to-hip ratio in predicting MetS.^[22]^

However, some studies have suggested that WHtR may not be superior as an indicator to other obesity indices. A meta-analysis in children and adolescents indicated that WHtR was not superior to BMI or WC in predicting cardiovascular risk factors.^[23]^ A study of 5429 Korean adults showed that both WHtR and WC had similar power for predicting cardiovascular risk factors,^[24]^ and other researchers suspected that the usefulness of WHtR was only due to its high correlation to WC.^[25]^ A study of Japanese workers also reported that WHtR was similar to BMI for predicating the clustering of cardiometabolic risk factors in its male-dominated study population.^[26]^ In our present study, we demonstrated that the AUC of the WHtR for predicting MetS was significantly higher than that of WC, at least in women. In men, the AUCs did not reach statistical significance, but the AUC of WHtR was still higher than that of WC. Therefore, it remains possible that WHtR may be more potent as a marker for predicting MetS in Asians.

The mechanism of the superiority of WHtR on predicting MetS might be that Asians have shorter statues than Caucasians and the use of WHtR takes into consideration the variation of height and it will be more accurate in representing central adipose tissue.^[8,9]^ Previous studies have shown that WHtR can be used to identify metabolic risks among Asian individuals classified as healthy, based on BMI and WC. Park and Kim^[27]^ demonstrated that in a Korean population with normal BMI and WC, the WHtR was still superior to BMI and WC in predicting MetS and its components (based on the 2005 IDF criteria). Similarly, Zhu et al^[28]^ identified the WHtR as a better screening tool to identify cardiometabolic risk factors in patients defined as normal according to BMI and WC, especially in women. Another cross-sectional study from Thailand also confirmed the usefulness of WHtR > 0.5 as a discriminating tool for CVD risk.^[29]^ More importantly, studies using WHtR also showed its application in predicting cardiometabolic risk factors in Caucasians,^[30,31]^ indicating that WHtR can be widely used. These studies also proposed a WHtR >0.5 as a cut-off point, which is closed to what we have demonstrated. Future studies with a prospective design and larger population will further accurately determine the WHtR cut-off value.

In our present study, we also considered a subgroup defined as not obese according to the BMI and WC criteria, and demonstrated that the ability of WHtR to discriminate remained true of this subgroup as well. This further confirms that BMI and WC may not suffice for evaluating the cardiometabolic risk of Chinese people. Adding WHtR as a new marker may identify more people who are potentially susceptible for cardiometabolic disease in Chinese populations.

Our results also indicated differences in the discriminating power of the obesity indices among the various metabolic disorders—hypertension, hyperglycemia, high TG, and low HDL-C. This is at variance with previous reports that showed that all obesity indices had similar power to assess cardiometabolic risks.^[32,33]^ However, a pooled analysis of 10 studies suggested that WHtR was the best discriminative marker for all MetS components.^[34]^ In the current study, our results indicated that WHtR was superior to BMI or WC in predicting hypertension and hyperglycemia, for both genders. However, with respect to high TG and low HDL-C, the discriminative power of WHtR, WC, and BMI was different. These data indicated that the discriminative power of WHtR is better for predicting hypertension and hyperglycemia, but not superior regarding dyslipidemia. These results require further study to verify.

In the present study, we found a potential interaction between age and gender and the obesity indices related to MetS. The AUCs of the obesity indices tended to decrease with age, suggesting that the discriminating power of the obesity indices for MetS was relatively weaker in the older group than in the younger group. A population-based study in Japan suggested that associations between obesity indices and cardiometabolic risks became weaker with increasing age.^[35]^ However, whether these data indicate that preventing obesity may be less effective in reducing the risk of CVD in the elderly requires further investigation.

Our study found that the optimal cutoff point of WHtR was similar for both genders. This is of interest, as both WC and height are regulated by sex steroids, and if our results are valid, then the use of WHtR may offset these gender differences. However, our results differ from previous studies that showed that men and women tended to have different WHtR cutoff values for predicting MetS.^[14,20,22]^ These gender differences need further validation.

The present study has several potential limitations. First, this is a cross-sectional design study, with its inherent limited interpretation of cause-and-effect temporality. Second, our study participants were selected from 1 urban district in China, and therefore, our results may not be representative for other populations. Third, we did not include hip circumference, and therefore, the waist-to-hip ratio was not included. Finally, the false-positive and false-negative values of WHtR, although lower than that of BMI and WC, were considered high, especially in the nonobese subpopulation. Therefore, prediction of MetS would require comprehensive evaluation other than anthropometric measurements. Cohort studies based on different ethnic populations are warranted to confirm our conclusions.

## 5 Conclusion

Our findings indicate that, in Han Chinese adults, the WHtR is a better predictor of MetS, and the components of MetS, than either WC or BMI. More importantly, the significant superiority of the WHtR remained true of the nonobese subgroup. Future prospective studies with a larger population can further validate the usefulness, as well as the limitations, of WHtR as a marker for MetS.

## Acknowledgment

The authors thank all participants involved in this study.

## Supplementary Material

Supplemental Digital Content
