# Supplementary material for: Waist-to-height ratio is better than body mass index and waist circumference as a screening criterion for metabolic syndrome in Han Chinese adults
Source: Medicine (Baltimore). 2017 Sep 29;96(39):e8192. doi: 10.1097/MD.0000000000008192 (PMC5626323; doi:10.1097/MD.0000000000008192)
Supplement: Supplemental Digital Content [file medi-96-e8192-s001.doc]

| **Table S1.** Partial correlation coefficients between obesity indices and metabolic disorders | | | | | | |
| --- | --- | --- | --- | --- | --- | --- |
|  | Women | | | Men | | |
| Variable | WHtR | WC | BMI | WHtR | WC | BMI |
| WHtR | — | 0.942 ^*^ | 0.782 ^*^ | — | 0.948 ^*^ | 0.842 ^*^ |
| WC | 0.942 ^*^ | — | 0.753 ^*^ | 0.948 ^*^ | — | 0.826 ^*^ |
| BMI | 0.782 ^*^ | 0.753 ^*^ | — | 0.842 ^*^ | 0.826 ^*^ | — |
| SBP | 0.280 ^*^ | 0.263 ^*^ | 0.277 ^*^ | 0.256 ^*^ | 0.242 ^*^ | 0.261 ^*^ |
| DBP | 0.309 ^*^ | 0.314 ^*^ | 0.350 ^*^ | 0.336 ^*^ | 0.333 ^*^ | 0.334 ^*^ |
| FPG | 0.119 ^*^ | 0.104 ^*^ | 0.078 ^*^ | 0.136 ^*^ | 0.122 ^*^ | 0.130 ^*^ |
| TG | 0.187 ^*^ | 0.188 ^*^ | 0.188 ^*^ | 0.278 ^*^ | 0.278 ^*^ | 0.271 ^*^ |
| HDL-C | –0.080 ^*^ | –0.085 ^*^ | –0.079 ^*^ | –0.140 ^*^ | –0.128 ^*^ | –0.112 ^*^ |
| LDL-C | 0.109 ^*^ | 0.096 ^*^ | 0.091 ^*^ | 0.252 ^*^ | 0.226 ^*^ | 0.221 ^*^ |
| TC | 0.104 ^*^ | 0.092 ^*^ | 0.099 ^*^ | 0.260 ^*^ | 0.238 ^*^ | 0.231 ^*^ |
| WHtR: waist-to-height ratio; WC: waist circumference; BMI: body mass index; SBP: systolic blood pressure; DBP: diastolic blood pressure; FPG: fasting plasma glucose; TG: triglycerides; HDL-C: high-density lipoprotein cholesterol  ^*^P < 0.05 for partial correlation analysis adjusted for age, education level, smoking, alcohol drinking, and physical exercise. | | | | | | |
